# The Role of Traditional Chinese Formula Ding-Kun Pill (DKP) in Expected Poor Ovarian Response Women (POSEIDON Group 4) Undergoing *In Vitro* Fertilization-Embryo Transfer: A Multicenter, Randomized, Double-Blind, Placebo-Controlled Trial

**DOI:** 10.3389/fendo.2021.675997

**Published:** 2021-06-17

**Authors:** Jing-Yan Song, Dan-Dan Gao, Xian-Ling Cao, Shan Xiang, Yan-Hua Chen, Yi-Li Teng, Xiu-Fang Li, Hai-Ping Liu, Fu-Xin Wang, Bin Zhang, Li-Hua Xu, Li Zhou, Xiang-Hong Huang, Zhen-Gao Sun

**Affiliations:** ^1^ The First Clinical College, Shandong University of Traditional Chinese Medicine, Jinan, China; ^2^ Reproductive and Genetic Center of Integrative Medicine, Affiliated Hospital of Shandong University of Traditional Chinese Medicine, Jinan, China; ^3^ College of Traditional Chinese Medicine, Shandong University of Traditional Chinese Medicine, Jinan, China; ^4^ Reproductive Medicine Center, Shanxi Maternal and Child Health Care Hospital, Taiyuan, China; ^5^ Reproductive Medicine Center, The Affiliated Hospital of Wenzhou Medical University, Wenzhou, China; ^6^ Center for Reproductive Medicine, Cheeloo College of Medicine, Shandong University, Jinan, China; ^7^ Key Laboratory of Reproductive Endocrinology of Ministry of Education, Shandong University, Jinan, China; ^8^ Department of Reproductive Medicine, The 960th Hospital of the PLA Joint Logistics Support Force Jinan, Jinan, China; ^9^ Center of Reproduction and Genetics, The Affiliated Suzhou Hospital of Nanjing Medical University, Suzhou, China; ^10^ Gusu School, Nanjing Medical University, Suzhou, China; ^11^ Department of Reproductive Medicine, The Second Affiliated Hospital of Shandong University of Traditional Chinese Medicine, Jinan, China; ^12^ Reproductive Medicine Center, Affiliated Hospital of Guangdong Medical University, Zhanjiang, China; ^13^ Department of Reproductive Medicine, The Reproductive Hospital of Guangxi Zhuang Autonomous Region, Nanning, China; ^14^ Reproduction & Genetics Center, Xiangtan Central Hospital, Xiangtan, China

**Keywords:** POSEIDON criteria, low prognosis, Ding-Kun Pill, traditional Chinese medicine, poor ovarian response, *in vitro* fertilization-embryo transfer

## Abstract

**Objective:**

The primary objective of the study was to assess traditional Chinese formula DKP supplementation in terms of efficacy and safety on reproductive outcomes of expected poor ovarian responder (POR, POSEIDON Group 4) undergoing *in vitro* fertilization-embryo transfer (IVF-ET).

**Design, Setting, and Participants:**

Women eligible for IVF-ET were invited to participate in this randomized, double-blind, placebo-controlled, superiority trial at academic fertility centers of ten public hospitals in Chinese Mainland. A total of 462 patients (35–44 years) equally divided between DKP and placebo groups with antral follicle count (AFC) <5 or anti-müllerian hormone (AMH) <1.2 ng/ml were randomized.

**Interventions:**

All participants were given DKP or 7 g placebo twice daily on the previous menstrual cycle day 5 until oocyte retrieval, which took approximately 5 to 6 weeks.

**Main Outcome Measure:**

The primary outcome was the ongoing pregnancy defined as more than 20 gestational weeks of an intrauterine living fetus confirmed by pelvic ultrasonography.

**Results:**

Demographic characteristics were equally distributed between the study populations. Intention-to-treat (ITT) analysis revealed that ongoing pregnancy rate (OPR) was not significantly different between DKP and placebo groups [26.4% (61/231) versus 24.2% (56/231); relative risk (RR) 1.09, 95% confidence interval (CI) 0.80 to 1.49, *P* = 0.593]. No significant differences between groups were observed for the secondary outcomes. The additional per protocol (PP) analysis was in line with ITT results: OPR in DKP group was 27.2% (61/224) versus 24.1% (55/228) in placebo group [RR 1.13, 95%CI (0.82 to 1.55), *P* = 0.449]. After subgroup analysis the findings concluded that POR population of 35–37 years had a significantly higher OPR after 5–6 weeks of oral DKP (41.8%, 33/79) versus placebo (25.4%, 18/71) [RR 1.65, 95% CI (1.02 to 2.65), *P* = 0.034, *P* for interaction = 0.028].

**Conclusion:**

This well-designed randomized controlled trial (RCT) offers new high-quality evidence to supplement existing retrospective literature concerning DKP performance in expected PORs. DKP could be recommended as a safe and natural remedy for expected PORs (aged 35–37 years) who fulfill the POSEIDON group 4 criteria. However, additional interventional clinical studies are undoubtedly required to be conducted in the future to validate this hypothesis.

**Clinical Trial Registration:**

www.chictr.org.cn, identifier ChiCTR1900026614.

## Introduction

The worldwide childbearing postponement is on the rise over the past few decades due to socioeconomic factors, such as accessibility to contraceptives, economic prosperity, improved education, and women’s workforce engagement (1, 2). This delay contributes to increased average age of the first attempt at conception, a proportional increase in women’s live births in their thirties, and higher pregnancy loss rates (3). The follow-through effect can be observed in the disproportionate use of assisted reproductive technology services among older women (4, 5). Besides, women are more vulnerable to decreased ovarian reserves (DOR) in their mid to late thirties associated with normal ovarian ageing, resulting in a growing number of older women having poor ovarian response (POR) during ovarian stimulation. This illustrates the necessity to devote significantly more attention to this women group undergoing *in vitro* fertilization-embryo transfer (IVF-ET) (6, 7).

The estimated POR prevalence ranges from 6 to 35%, which poses a severe challenge in assisted reproductive technology (8, 9). Moreover, large discrepancies in POR definition exist in preceding studies (10). This lack of uniformity resulted in the Bologna criteria in 2011, exemplifying the first significant attempt to establish specific POR definition standards (11). However, the Bologna criteria were questioned because of persistent heterogeneity among POR patients and the inability to provide management strategies (12, 13). Given the above-mentioned facts, more recent criteria, the POSEIDON classification, suggesting a new concept of low prognosis, was developed to provide a homogeneous and refined POR definition, resulting in significant heterogeneity reduction in Bologna POR population and individualized treatment promotion in these patients (14).

Among all POSEIDON groups, group 4 (age ≥35 years and AFC <5 or AMH <1.2 ng/ml) has been estimated to constitute 55% of patients (15). POSEIDON group 4 subpopulations have a considerably lower prognosis due to age-related increase in oocyte euploidy, leading to more aneuploid embryos and higher ET cancellation rates. Managing such patients is a daunting task; however, the treatment objective is to enhance the probability of producing at least one euploid blastocyst to be transferred to the individual patient. Although more evidence is required, this might be accomplished, possibly by adding adjuvant treatment to ovarian stimulation (OS) protocol or before OS initiation (15, 16). Regardless of the various pre-treatment strategies, comprising coenzyme Q10 and dehydroepiandrosterone (DHEA), insufficient evidence is found on the efficacy of these therapeutic agents to reverse low prognosis, particularly in women with advanced reproductive age or DOR (17–19).

DKP is one of the famous traditional Chinese medicine prescriptions that was first utilized during Emperor Qianlong’s reign of Qing dynasty (A.D. 1636–1912) as a unique formula in the emperor’s harem, accompanied by exclusive utilization by the imperial court. The approved DKP formula empowered few companies to produce, such as Shanxi Guangyuyuan Traditional Chinese Medicine Co., Ltd., whose DKP was rated as National intangible cultural heritage by the State Council of the People’s Republic of China in 2011. DKP components comprise ginseng, deer antler, safflower, angelica, scutellaria, rhizoma cyperi, ligustrazine, and other 30 precious Chinese herbal and animal orient medicine. Based on traditional Chinese medicine (TCM) theory, DKP has been deployed as a blood-activating and Qi-nourishing formula toward improving and curing several prevalent gynecological diseases, such as menstrual disorders, dysmenorrhea, menopausal syndrome and other physical symptoms (20). Meanwhile, modern pharmacological studies indicated that DKP could decrease blood viscosity, plasma viscosity and hematocrit, enhance inflammation and hypoxia, and promote mice’s uterus development (21). In clinical practice of Chinese medicine, TCM pathogenesis in elderly women with low prognosis is mainly manifested by spleen and kidney deficiency, blood deficiency, and liver depression, consistent with TCM syndrome type of DKP. Moreover, no RCTs have investigated DKP supplementation effectiveness based on POSEIDON stratification in IVF cycles so far. In our previous prospective cohort study, we observed that older women with low prognosis following DKP pretreatment had more oocytes and embryos than those without DKP intervention. Although DKP group had a higher clinical pregnancy and ongoing pregnancy rates, yet the difference was not statistically significant (22).

As a consequence, such a well-designed randomized controlled trial devotes to investigating efficacy and safety of DKP on reproductive outcomes of IVF-ET in women with low prognosis who meet the POSEIDON group 4 criteria.

## Materials and Methods

### Design and Participants

The study design was a multicenter, randomized, double-blinded, placebo-controlled, superiority trial with a 1:1 allocation to either DKP or placebo groups. Following the study approval by ethics committees of participating hospitals, all couples provided voluntary written informed consent prior to participation. A data and safety monitoring board have been established to manage the study. The study rationale and a detailed trial protocol have been published elsewhere previously (23). The current study followed the Consolidated Standards of Reporting Trials (CONSORT) reporting guideline.

Participants eligible for RCT underwent IVF/intracytoplasmic sperm injection (ICSI) cycles and fulfilled POSEIDON group 4 stratification based on the Bologna criteria. POSEIDON group 4 is known as ≥35 years old with poor pre-stimulation ovarian reserve parameters (AFC <5 or AMH <1.2 ng/ml) and with an expected poor ovarian response (fewer than four oocytes) after standard ovarian stimulation. The exclusion criteria were as follows: (i) Individuals with a Body Mass Index (BMI) ≥30 kg/m^2^; (ii) Those using the natural cycle or mild stimulation for IVF/ICSI treatment; (iii) Those with a history of unilateral oophorectomy or recurrent pregnancy loss, defined as two or more spontaneous abortions; (iv) Acceptors of donated oocytes or performed either In vitro Maturation (IVM) or blastocyst biopsy for Preimplantation Genetic Diagnosis (PGD) or Preimplantation Genetic Testing for Aneuploidies (PGT-A); (v) Those previously diagnosed with congenital (e.g., mediastinal uterus and double uterus) or acquired (e.g., submucosal myoma and adenomyosis) uterine abnormalities; (vi) Patients with extremely advanced age (≥45 years old); and (vii) Presence of a non-surgically treated hydrosalpinx or endometrial polyp and an ovarian endometriosis cyst requiring surgery, during ovarian stimulation.

### Randomization and Blinding

Eligible participants were invited to enroll in RCT by advertisement, and they were recruited from November 15, 2019 to July 7, 2020. A total of 462 couples were randomly allocated in four blocks into either DKP or placebo groups using a computerized random number generator (R 4.0.0, R Foundation for Statistical Computing, Vienna, Austria), ensuring a 1:1 allocation ratio. Therefore, each block resulted in allocating four patients to each group. A study staff generated the sequences and assigned the participants to DKP and placebo groups without taking part in intervention delivery, data collection, or data analysis. Participants were enrolled by staff without involving in randomization process. Both medications (DKP formula and placebo) were prepared with identical shape, taste, and smell.

### Treatment Procedures

#### DKP Formula and Placebo Preparation

The decoction is generally prepared by boiling in water for hours. However, DKP (Lot No. 3271911068, Shanxi Guangyuyuan Traditional Chinese Medicine Co., Ltd, Shanxi, China) was prepared by adopting water-honeyed pill protocol according to Chinese Pharmacopoeia (ChP) 2015 Edition standard. The “DKP water-honeyed pill” standard is approved by the China Food and Drug Administration (CFDA). Each bottle is filled with 7 g DKP.

DKP is mainly composed of the following 30 medicinal herbs, including Radix Ginseng, Cornu Cervi Pantotrichum, Radix Angelicae sinensis, Radix Rehmanniae Preparata, Stigma Croci, Caulis Spatholobi, Radix Notoginseng, Radix Paeoniae Alba, Rhizoma Atractylodis Macrocephalae, Fructus Lycii, Radix Scutellariae, Rhizoma Cyperi, Fructus Leonuri, Rhizoma Ligustici Chuanxiong, Cornu Cervi Degelatinatum, Colla Corii Asini, Rhizoma Corydalis, Flos Carthami, Herba Leonuri, Faeces Togopteri, Poria, Radix Bupleuri, Radix Linderae, Fructus Amomi Villosi, Cortex Eucommiae, Rhizoma Zingiberis, Herba Asari, Radix Cyathulae, Cortex Cinnamomi, and Radix Glycyrrhizae.

The genuine medicinal materials are used in all kinds of traditional Chinese herbal and animal medicines, and specific purchasing locations are stipulated as township-level sales points where genuine medicinal materials are located and purchased in the same batch. The medicinal materials are processed according to requirements, and standard operating procedures are formulated. The quality control results of DKP were consistent with Chinese Medicine Standards of State Food and Drug Administration (SFDA) (24, 25).

The placebo is provided by Shanxi Guangyuyuan Traditional Chinese Medicine Co. Ltd. (China) as a mixture of 55% starch and 45% caramel that were mixed, dried, crushed, and lumped together. The daily placebo doses are packed in individual bottles for easy consumption under ChP 2015 Edition standard, Good Manufacture Practice of Medical Products (GMP) standard. Patients in placebo group consume the same amount of placebo as treatment group. The placebo and Chinese medicines were used to make DKP identical in appearance, color, smell, taste, packaging, usage, and dosage (26). During placebo production, selecting condiments, colorants, and other excipients should be carefully carried out and strictly in accordance with Chinese Medicine Standards of SFDA.

Before ovarian stimulation, all participants can obtain DKP or 7 g placebo orally twice daily for approximately 5 to 6 weeks, from day 5 of the previous menstrual cycle until oocyte recovery.

#### Ovarian Stimulation Regimen

All participants started ovarian stimulation with a Gonadotropin-releasing hormone (GnRH) antagonist regimen on menstrual cycle day 2 or 3. GnRH antagonist (0.25 mg, cetrorelix; Merck Serono, Darmstadt, Germany) was administered subcutaneously at a daily dose of 0.25 mg when there is at least one follicle measuring ≥12 mm in mean diameter, with 150–300 IU/day of recombinant follicle-stimulating hormone (rFSH) (75IU, Puregon, MSD, Courbevoie, France; Gonal-F, Merck-Serono, Lyon, Italy) and recombinant luteinizing hormone (rLH) (75IU, Luveris^®^, Merck-Serono, Germany). Gonadotropin doses were determined based on individual patient’s characteristics. Final oocyte maturation must be triggered when more than one leading follicle measuring 18 mm or greater are visible by ultrasound. Final oocyte maturation needed to be achieved by both 0.2 mg injection of GnRH agonist (0.1 mg, Triptoreline, Decapeptyl, Ipsen, France) and 250 μg of recombinant human chorionic gonadotropin (rhCG, 250 μg, Ovitrelle, Serono, France) (27). Oocyte retrieval was accomplished by transvaginal ultrasound-guided aspiration after 34–35 h.

#### Oocyte Retrieval and Embryo Culture

BD Falcon IVF medium (Becton, Dickinson and Company, Franklin Lakes, NJ, USA) was employed to collect oocytes and perform embryo culture. Incubation conditions were set at 6% CO_2_, 5% O_2_, and 37.0°C (C200 CO_2_ Incubator, Labotect Labor-Technik-Göttingen GmbH, Göttingen, Germany). Cultures oocytes were inseminated for IVF or decumulated for ICSI.

Transfer of one or two high-quality embryos was performed on day 3 or 5, and the surplus was frozen on same days based on routine at various sites. All good quality embryos were cryopreserved *via* vitrification (CBS-ViT-HS, CryoBioSystem^®^, L’Aigle, France). Dimethylsulfoxide and ethylene glycol were used as cryoprotectants (Irvine Scientific Freeze Kit^®^, Irvine Scientific, Newtown Mount Kennedy, Ireland and Vitrification Kit 101, Cryotech^®^, Tokyo, Japan).

#### Endometrial Preparation and Embryo Transfer

For patients assigned to fresh embryo transfer, intramuscular progesterone at a daily dose of 60 mg was administered for luteal-phase support, beginning on oocyte retrieval day until 8–10 weeks after conception. For patients assigned to frozen–thawed embryo transfer (FET), no luteal-phase support was administered after oocyte retrieval, and day-3 embryos or day-5 blastocysts were cryopreserved for later transfer.

Artificial endometrial preparation consisted of sequential administration of E_2_ valerate and intramuscular progesterone. A total of 2 mg E_2_ valerate were administered twice daily for 6–8 days, and the dose was later adjusted based on endometrial thickness measured by vaginal ultrasonography. For endometrial thickness ≥7 mm, intramuscular progesterone 60 mg was initiated, while for endometrial thickness <7 mm, the patients continued taking oral E_2_ until the endometrium attained the required threshold. On day 4 or 6 of progesterone regimen, one or two day-3 frozen embryos or day-5 blastocysts were thawed and transferred. Ultrasound-guided soft catheter embryo transfers were performed. Once pregnancy was confirmed 14 days after FET, the luteal-phase support with estradiol valerate and intramuscular progesterone for endometrial preparation continued until 8–10 weeks of gestation.

### Study Outcomes

This work’s primary outcome was ongoing pregnancy rate per randomized patient, which also included natural pregnancies. We defined ongoing pregnancy as a detectable fetal heartbeat after 20 weeks of gestation. Ongoing pregnancy rate was recorded per randomized patient, started stimulation, oocyte retrieval, and embryo transfer. Secondary outcomes were positive pregnancy rates (biochemical pregnancies), embryo implantation rates, clinical pregnancy rates, ectopic pregnancy rates, pregnancy loss rates, and twin pregnancies, including women admitted to hospital for abnormal pregnancies. Definitions for secondary outcomes are provided in **Supplementary Table 2**.

### Statistical Analysis

We designed the trial as a superiority study using PASS software version 11.0 (NCSS, LLC. Kaysville, Utah, USA) to calculate sample size. Sample size calculation indicated that at least 203 patients in each group were required to have a 90% power at a significance level of 0.05 to detect an absolute difference of 15% in the ongoing pregnancy rate with DKP supplementation, with an estimated rate of 25% in Placebo group. The effect size of 15% was based on existing scarce literature. Our previous study found a difference of 14.4% in the ongoing pregnancy rate for DKP group compared with non-DKP one (22). Therefore, the effect size of 15% was based on these limited numbers; however, the trial plans to include 462 participants, with 231 in each arm, to account for an expected 10% loss to follow-up.

**Figure 1:**
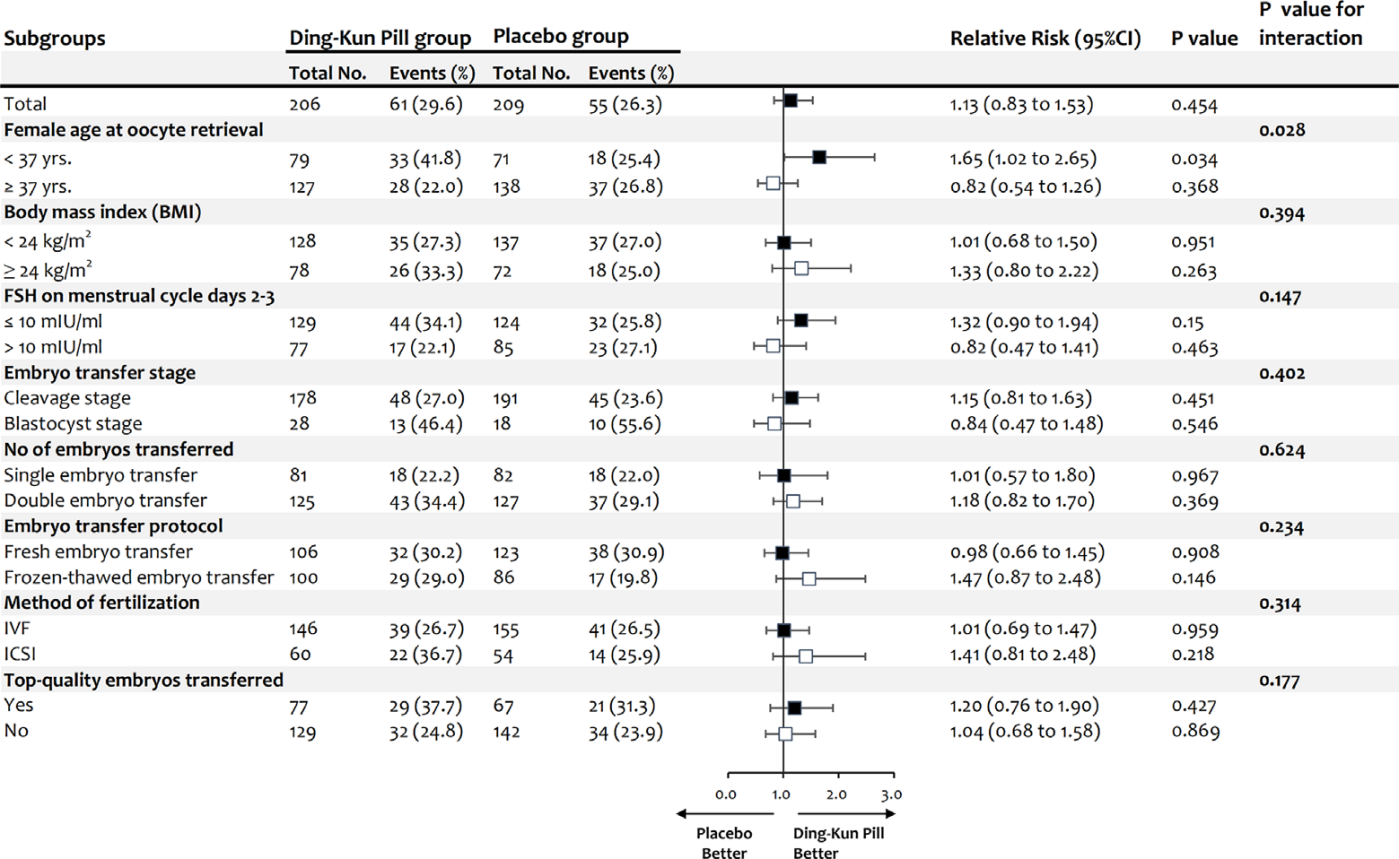
Subgroup analysis of ongoing pregnancy rate per embryo transfer for women in Ding-Kun Pill and placebo groups. (FSH, follicle stimulating hormone; IVF, *in vitro* fertilization; ICSI, intracytoplasmic sperm injection).

We used the intention-to-treat principle for the primary statistical analysis. Primary and secondary outcomes were assessed by comparing the outcome after the first embryo transfer. All women were accounted for in the group to which they were randomized, regardless of whether they received the prespecified treatment. We included all women who adhered strictly to the study protocol in a *post hoc* per protocol and subgroup analyses (**Figure 1** and **Supplementary Table 1**). We determined ongoing pregnancy rate, and relative risk was used to describe the difference. We compared continuous data utilizing a Student t-test or a Wilcoxon rank-sum test, and the results are given as mean (SD, standard deviation) or median (IQR, interquartile range). Categorical data were assessed using chi-square analysis and Fisher’s exact test for expected frequencies less than 5. A two-sided P value of <0.05 was considered to indicate statistical significance. All analyses were performed using SPSS version 26.0 and R statistical package version 4.0.0.

## Results

### Study Patients

**Figure 2:**
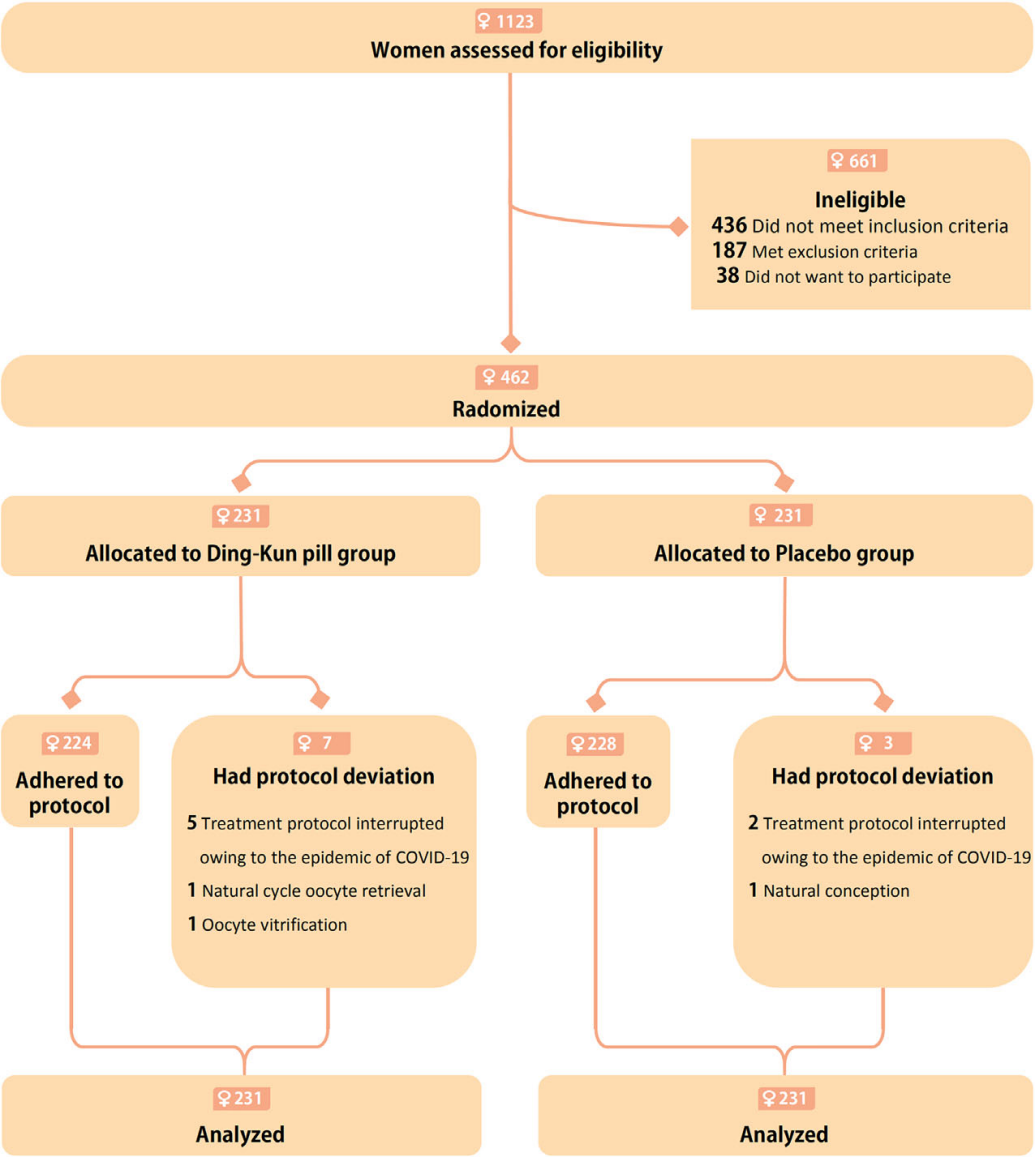
Flow chart depicting the randomized assignment of women to the Ding-Kun Pill and Placebo groups, exclusions, and protocol deviations.

The baseline demographics and clinical characteristics of patients were comparable between the study groups (**Table 1**). A total of 10 patients deviated from the protocol, including seven of 231 (3.0%) in DKP group and three of 231 (1.3%) in placebo group (**Figure 2**). Of these women, 453 had oocytes retrieved: 225 (97.4%) in DKP group and 228 (98.7%) placebo group. After ovarian stimulation, four (1.7%) in DKP group and eight (3.5%) in placebo group had no oocytes retrieved (**Table 2**). Additionally, 14 women (6.1%) in DKP group and nine (3.9%) in placebo group did not have an embryo available for transfer (**Table 2**). Two (0.9%) women in placebo group did not have a blastocyst for transfer, and one woman (0.4%) in DKP group had all oocytes frozen (**Table 2**).

**Table 1:** Participants’ baseline characteristics on menstrual cycle days 2–3.

Characteristics	Ding-Kun Pill group (n = 231)	Placebo group (n = 231)	P value
Age at inclusion (years; mean (SD)):	37.9 (2.3)	37.8 (2.2)	0.771
Age ≥37	147 (63.6)	155 (67.1)	0.434
Age ≥40	67 (29.0)	58 (25.1)	0.346
Body mass index (kg/m^2^; mean (SD)) ⁑	23.2 (3.0)	23.0 (2.9)	0.372
Duration of infertility (years; mean (IQR))	3.3 (4.0)	4.0 (4.0)	0.307
Nulliparous	68 (29.4)	82 (35.5)	0.164
Primary cause of infertility:			0.816
Tubal factor	168 (72.7)	159 (68.8)	
Male factor	52 (22.5)	59 (25.5)	
Tubal + Male factor	7 (3.0)	9 (3.9)	
Unexplained infertility	4 (1.7)	4 (1.7)	
AMH (ng/ml; median (IQR))	0.9 (0.6)	0.9 (0.6)	0.677
Total AFC (mean (IQR))	5.0 (2.0)	5.0 (2.0)	0.561
FSH (mIU/ml; mean (IQR)) 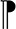	9.0 (5.1)	9.0 (5.2)	0.795
LH (mIU/ml; mean (IQR)) 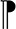	4.2 (3.3)	4.6 (3.0)	0.128
Estradiol (pg/ml; mean (IQR)) 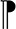	46.3 (30.0)	50.1 (30.0)	0.367

In any of the baseline characteristics, no significant differences between groups (P <0.05) were observed. AMH, anti-müllerian hormone; AFC, antral follicle count; FSH, follicle stimulating hormone; LH, luteinizing hormone; IQR, interquartile range; SD, standard deviation.

⁑Body mass index is weight (kg) divided by height squared (m^2^).

^
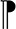
^FSH was missing for two women in placebo group. LH was missing for one woman in Ding-Kun Pill group and for three women in placebo group. Estradiol was missing for one woman in Ding-Kun Pill group and for three women in placebo group.

Data are presented as numbers (%) unless otherwise noted.

**Table 2:** Controlled ovarian stimulation and *in vitro* fertilization-embryo transfer characteristics in study population.

Characteristics	Ding-Kun Pill group (n = 231)	Placebo group (n = 231)	P value
No. of days of COS (mean (SD))	9.6 (2.4)	9.5 (2.4)	0.504
Total gonadotrophin dose administered (IU; mean (IQR))	2,100 (2,325)	2,025 (1,350)	0.269
Estradiol on hCG trigger day (pg/ml; mean (IQR))	1,214.6 (857.3)	1,281 (999)	0.465
Progesterone on hCG trigger day (ng/ml; mean (IQR))	0.6 (0.6)	0.7 (0.5)	0.427
Method of fertilization:			0.435
IVF	162/224 (72.3)	170/225 (75.6)	
ICSI	62/224 (27.7)	55/225 (24.4)	
No. of oocytes retrieved (median (IQR))	6.0 (3.0)	6.5 (4.0)	0.855
No. of two PN oocytes (fertilized; median (IQR)) †	5.0 (3.0)	4.0 (5.0)	0.383
No. of two PN cleavage zygotes (median (IQR))	5.0 (4.0)	4.0 (4.0)	0.279
No. of embryos available for transfer (median (IQR))	3.0 (3.0)	4.0 (3.0)	0.265
No. of high-quality day 3 embryos (median (IQR)) 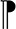	2.0 (2.0)	2.0 (2.0)	0.355
No. of high-quality blastocysts (median (IQR)) ‡	2.0 (1.0)	1.0 (0)	0.014
No. of embryos transferred (mean (IQR)):	2.0 (1.0)	2.0 (1.0)	0.983
Single embryo transfer	81/206 (39.3)	82/209 (39.2)	0.986
Double embryo transfer	125/206 (60.7)	127/209 (60.8)	0.986
Embryo transfer stage:			0.106
Cleavage stage	178/206 (86.4)	191/209 (91.4)	
Blastocyst stage	28/206 (13.6)	18/209 (8.6)	
Fresh embryo transfer	102/202 (50.5)	121/207 (58.5)	0.106
Endometrial thickness on hCG trigger day (mm; mean (SD))	9.8 (1.8)	9.9 (2.3)	0.839
Frozen-thawed embryo transfer	100/202 (49.5)	86/207 (41.5)	0.106
Endometrial thickness prior to FET (mm; mean (SD)) §	9.1 (1.4)	9.2 (1.7)	0.877
No. of women with no oocytes retrieved after COS	4/225 (1.8)	8/228 (3.5)	0.264
No. of women with no embryo transfer after aspiration:			0.169
No blastocyst development	0/224	2/225	
No day-3 embryo available for transfer	14/224	9/225	
Oocyte vitrification	1/224	0/225	

COS, controlled ovarian stimulation; hCG, human chorionic gonadotropin; IVF, *in vitro* fertilization; ICSI, intracytoplasmic sperm injection; IQR, interquartile range; SD, standard deviation.

^†^Two distinct pronuclei defined by four cells, a maximum of 10% fragmentation, and no multinucleation.

^
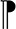
^Typically, a good, normally growing day 3 embryos will contain between six and 10 cells.

^‡^Defined as Gardner score 3BB or higher.

^§^Programmed cycle defined by administration of both estradiol and progesterone.

Data are number/total number or number (%) unless stated otherwise.

### COS and IVF Characteristics

**Table 2** shows the characteristics of enrolled women according to COS and IVF procedures. No significant differences were found in most COS and IVF outcomes between DKP and placebo groups. Conversely, median number of high-quality blastocysts was significantly higher in DKP group (2.0, IQR 1.0) compared with placebo group (1.0, IQR 0; P = 0.014).

### Ongoing Pregnancy and Secondary Outcomes

The primary analysis was performed according to the intention-to-treat principle. In DKP group, 61 of 223 women (26.4%) had an ongoing pregnancy compared with 56 of 231 (24.2%) in placebo group, for a relative risk (RR) of 1.09 [95% confidence interval (CI), 0.80 to 1.49; P = 0.593; **Table 3**]. No significant difference was found in clinical pregnancy rate between DKP and placebo groups [77 of 231 (33.3%) and 70 of 231 (30.3%), respectively; RR 1.10, 95% CI 0.84 to 1.44, P = 0.484; **Table 3**]. One woman in placebo group conceived naturally before oocyte retrieval (**Figure 2**). No significant difference was present in embryo implantation rate, ectopic pregnancy rate, pregnancy loss rate, and twin pregnancies between the two groups.

**Table 3:** Reproductive outcomes for women in Ding-Kun Pill and placebo groups (intention-to-treat analysis).

Outcomes	Ding-Kun Pill group (n = 231)	Placebo group (n = 231)	Relative risk (95% CI)	P value
**Primary outcome**				
Ongoing pregnancy *				
Ongoing pregnancy rate/No. of randomised women	61/231 (26.4)	56/231 (24.2)	1.09 (0.80 to 1.49)	0.593
Ongoing pregnancy rate/No. of women who started stimulation	61/225 (27.1)	55/228 (24.1)	1.12 (0.82 to 1.54)	0.466
Ongoing pregnancy rate/No. of oocyte retrievals	61/225 (27.1)	55/225 (24.4)	1.11 (0.81 to 1.52)	0.518
Ongoing pregnancy rate/No. of embryo transfers	61/206 (29.6)	55/209 (26.3)	1.13 (0.83 to 1.53)	0.454
**Secondary outcomes**				
Clinical pregnancy				
Clinical pregnancy rate/No. of randomised women	77/231 (33.3)	70/231 (30.3)	1.10 (0.84 to 1.44)	0.484
Clinical pregnancy rate/No. of women who started stimulation	77/225 (34.2)	69/228 (30.3)	1.13 (0.87 to 1.48)	0.367
Clinical pregnancy rate/No. of oocyte retrievals	77/225 (34.2)	69/225 (30.7)	1.12 (0.85 to 1.46)	0.421
Clinical pregnancy rate/No. of embryo transfers	77/206 (37.4)	69/209 (33.0)	1.21 (0.81 to 1.81)	0.352
Positive pregnancy †				
Positive pregnancy rate/No. of randomised women	85/231 (36.8)	82/231 (35.5)	1.04 (0.81 to 1.32)	0.771
Positive pregnancy rate/No. of women who started stimulation	85/225 (37.8)	81/228 (35.5)	1.06 (0.84 to 1.36)	0.619
Positive pregnancy rate/No. of oocyte retrievals	85/225 (37.7)	81/225 (36.0)	1.05 (0.74 to 1.58)	0.696
Positive pregnancy rate/No. of embryo transfers	85/206 (41.3)	81/209 (38.8)	1.07 (0.84 to 1.35)	0.602
Pregnancy loss rate ‡	38/85 (44.7)	36/82 (43.9)	1.02 (0.73 to 1.43)	0.917
Pregnancy loss ≤12 weeks of gestation	37/85 (43.5)	34/82 (41.5)	1.05 (0.74 to 1.50)	0.787
Pregnancy loss >12 weeks of gestation	1/85 (1.2)	2/82 (2.4)	0.48 (0.05 to 5.22)	0.616
Ectopic pregnancies ‡	0/85 (0)	1/82 (1.2)	–	0.491
Embryo implantation rate (median (IQR)) 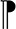	0 (0.5)	0 (0.5)	–	0.500
Twin pregnancies	5/77 (6.5)	11/70 (15.7)	0.41 (0.15 to 1.13)	0.073

All analyses by intention to treat.

*Ongoing pregnancy was defined as a detectable fetal heart beat after 20 weeks of gestation.

^†^Positive pregnancy (biochemical pregnancy), i.e. serum β-hCG level ≥10 mIU/ml.

^‡^Denominator defined as number of positive β-hCG values (≥10 IU/ml) in each group.

^
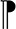
^Embryo implantation rate was defined as the number of intrauterine gestational sacs observed divided by the number of embryos transferred.

Data are number/total number (%) of women unless stated otherwise.

The frequency of positive pregnancy, clinical pregnancy, and ongoing pregnancy per embryo transfer was not significantly different between DKP and placebo groups. The ongoing pregnancy rate per embryo transfer was 29.6% (61/206) versus 26.3% (55/209) in DKP and placebo groups, respectively (RR 1.13, 95% CI 0.83 to 1.53, P = 0.454; **Table 3**). The per-protocol analysis results were consistent with intention-to-treat analysis results, as displayed in **Supplementary Table 1**.

### Subgroup Analysis of Ongoing Pregnancy Rate Per Embryo Transfer

The results were similar across most subgroups; nevertheless, the advantage of DKP supplementation tended to be more pronounced among patients younger than 37 years than in elderly patients between 37 and 44 years [33/79 (41.8%) versus 18/71 (25.4%); RR 1.65, 95 CI 1.02 to 2.65; P = 0.034; P = 0.028 for interaction] (**Figure 1** and **Supplementary Table 3**).

### Safety Assessment

No adverse events occurred in either DKP group versus placebo group. Safety indicators of complete blood cell count and liver and kidney function before and after treatment were within reasonable limits in both groups.

## Discussion

To the best of our knowledge, this is the largest RCT performed to evaluate DKP supplementation effects on reproductive outcomes in patients undergoing IVF-ET who fulfilled POSEIDON group 4 criteria. In this multicenter, randomized controlled trial, we found no significant differences in ongoing pregnancy and clinical pregnancy rates between DKP and placebo groups. However, DKP supplementation resulted in a significantly higher number of high-quality blastocysts compared with placebo group. Besides, the benefit of utilizing DKP seemed to be more apparent in patients below 37 years than in ones between 37 and 44 years.

According to TCM principle, POR belongs to categories of ‘infertility’, ‘hypomenorrhea’, ‘amenorrhea’ and ‘menopausal syndrome’. In TCM, POR is associated with spleen and kidney deficiency, liver depression and blood vacuity (28, 29). The Yellow Emperors Internal Classic, a famous book published more than 2,000 years ago, states that females’ basic physiological processes are linked to kidneys. Besides, stagnation of liver and spleen are thought to be responsible for POR (30). Accordingly, improving the physical condition of kidney, liver and spleen may improve POR. The present study investigated DKP effect on POR who meet the POSEIDON group 4 stratification. The DKP is derived from ‘Si Wu’ decoction, ‘Si Jun Zi’ decoction, and ‘Chaihu Shugan’ powder and includes more than 30 types of Chinese herbal and animal medicines. In TCM, this mixture is assumed to tonify the kidney, invigorate blood circulation, smooth the liver and invigorate the spleen. As a result, it was hypothesized that DKP may be beneficial for POR patients.

Actually, DKP has been used in polycystic ovarian syndrome (PCOS) with concomitant ovulatory defects, insulin resistance, and menstrual abnormalities, although clinical trials utilizing DKP in POR population are quite scarce (31, 32). Two randomized controlled trials (RCTs) have recently investigated DKP supplementation’s role in POR therapy (30, 33). Xie discovered that using DKP and clomiphene in treating patients with reduced ovarian reserve substantially raised FSH, AFC, AMH, and estradiol levels as opposed to clomiphene alone. Moreover, their life quality, ovulation rate, and clinical pregnancy rate all increased dramatically (P <0.01) (33). However, several variables render it impossible to reliably randomize participants, and since this was performed in an outpatient clinic, the results made cannot be verified and have little particular scientific value for women undergoing IVF-ET. In patients with a poor response to OS, Wei and his colleagues evaluated the impact of DKP and micro ovarian stimulation on clinical outcomes. The findings showed that compared to non-DKP group, DKP significantly increased estradiol concentration and endometrial thickness on hCG trigger day, decreased Gn dose, duration and cycle cancellation rate, and increased numbers of oocytes retrieved, high-quality embryos, embryo implantation rate, and clinical pregnancy rate (30). The study described above had clear randomization but not blinding and allocation concealment; meanwhile, the participants’ recruitment process was unclear, and the sample size estimate was uncertain. Additionally, the inclusion criteria were introduced by the 2012 Bologna Consensus, but eligible participants were not stringent, and there was considerable variability between populations (30). Fortunately, DKP pretreatment promotes ovarian sensitivity to exogenous gonadotropins in POR patients, but this may contribute to greater oocyte developmental capacity, greater endometrial receptivity, and a higher clinical pregnancy rate, all of which allows DKP to have certain benefit.

The detailed molecular mechanisms of the effect of DKP on oocytes, cumulus cells, and granulosa cells are still unclear. A recent experimental study found that DKP can effectively activate the implantation rate of delayed embryo implantation mouse model by regulating the genes related to ‘endometrium-embryo interface’ (34). Moreover, according to Ma et al., it was demonstrated that DKP was able to increase ovarian reserves through inhibiting PI3K/AKT/mTOR signaling pathway, leading to suppression of primordial follicle activity and a reduction in levels of apoptosis of early growing follicles (35). All in all, these findings demonstrate the potentially beneficial role of DKP in treating DOR or POR. However, further studies are required to explore the molecular mechanisms underlying DKP actions.

Our trial is the first and largest multicenter randomized placebo-controlled trial to date to investigate DKP supplementation impact in POR patients. Moreover, we performed randomization at baseline on menstrual cycle day 2 or 3. This approach ensured minimal selection bias in the women included in our study and not only those with more oocytes retrieved after ovarian stimulation. Furthermore, low prognosis patients were classified by POSEIDON groups which significantly reduced the heterogeneity identified in Bologna POR population. The same dosage of DKP or placebo was used in all patients treated. Our study has some limitations. Based on our previous study, even though we have prolonged the DKP period of intervention in patients with POR, we must conduct more studies on the optimum DKP treatment duration in the future (22). Moreover, the superiority study design had the power to detect a 15% difference in ongoing pregnancy rate between the two groups; therefore, smaller but clinically important differences might be overlooked.

## Conclusions

In conclusion, DKP pretreatment in an IVF/ICSI cycle can improve the number of high-quality blastocysts in patients who accomplish the POSEIDON group 4 criteria. Moreover, DKP supplementation may raise OPR, especially in patients younger than 37 years. However, larger, well-designed interventional studies are required to further demonstrate the clinical relevance of DKP on improving reproductive outcomes for these subpopulations.

## Data Availability Statement

The raw data supporting the conclusions of this article will be made available by the authors, without undue reservation.

## Ethics Statement

The studies involving human participants were reviewed and approved by the Health Authorities and Ethics Committees of the Affiliated Hospital of Shandong University of TCM. The patients/participants provided their written informed consent to participate in this study. Written informed consent was obtained from the individual(s) for the publication of any potentially identifiable images or data included in this article.

## Author Contributions

Z-GS had full access to all data in the study and takes responsibility for data integrity and data analysis accuracy. Concept and design: J-YS and Z-GS. Acquisition, analysis, or interpretation of data: D-DG, X-LC, and SX. Drafting of the manuscript: J-YS. Critical revision of the manuscript for important intellectual content: Y-HC, Y-LT, X-FL, and H-PL. Statistical analysis: F-XW, BZ, L-HX, LZ, and X-HH. Supervision: Z-GS and Y-LT. All authors contributed to the article and approved the submitted version.

## Funding

This research was mainly supported by the Key Technology R&D Program of Shandong Province (No. 2017G006016).

## Conflict of Interest

The authors declare that the research was conducted in the absence of any commercial or financial relationships that could be construed as a potential conflict of interest.

## Supplementary Material

Supplementary Figure 1 Dingkun Pill, a traditional Chinese medicine with a long history.

## References

[1] Leridon H. Demographic Effects of the Introduction of Steroid Contraception in Developed Countries. Hum Reprod Update (2006) 12(5):603–16. 10.1093/humupd/dml025 16775191

[2] ESHRE Guideline Group on Ovarian Stimulation Bosch E Broer S Griesinger G Grynberg M Humaidan P. Erratum: ESHRE Guideline: Ovarian Stimulation for IVF/ICSI. Hum Reprod Open (2020) 2020(4):hoaa067. 10.1093/hropen/hoaa067 33409381 PMC7770487

[3] Lutz W O’Neill BC Scherbov S. Demographics. Europe’s Population At a Turning Point. Science (2003) 299(5615):1991–2. 10.1126/science.1080316 12663901

[4] Adamson GD de Mouzon J Chambers GM Zegers-Hochschild F Mansour R Ishihara O. International Committee for Monitoring Assisted Reproductive Technology: World Report on Assisted Reproductive Technology, 2011. Fertil Steril (2018) 110(6):1067–80. 10.1016/j.fertnstert.2018.06.039 30396551

[5] Meczekalski B Szeliga A Podfigurna A Miechowicz I Adashi EY. Assisted Reproductive Technology Outcome in United States of America and Australia With New Zealand: Comparison of Annual Reports 2005-2016. Gynecol Endocrinol (2020) 36(11):959–67. 10.1080/09513590.2020.1737006 32172637

[6] Cohen Y Tannus S Alzawawi N Son WY Dahan M Buckett W. Poor Ovarian Response as a Predictor for Live Birth in Older Women Undergoing IVF. Reprod BioMed Online (2018) 36(4):435–41. 10.1016/j.rbmo.2018.01.008 29478839

[7] Kocourkova J Burcin B Kucera T. Demographic Relevancy of Increased Use of Assisted Reproduction in European Countries. Reprod Health (2014) 11:37. 10.1186/1742-4755-11-37 24885428 PMC4049397

[8] Patrizio P Vaiarelli A Levi Setti PE Tobler KJ Shoham G Leong M. How to Define, Diagnose and Treat Poor Responders? Responses From a Worldwide Survey of IVF Clinics. Reprod BioMed Online (2015) 30(6):581–92. 10.1016/j.rbmo.2015.03.002 25892496

[9] Oudendijk JF Yarde F Eijkemans MJ Broekmans FJ Broer SL. The Poor Responder in IVF: Is the Prognosis Always Poor?: A Systematic Review. Hum Reprod Update (2012) 18(1):1–11. 10.1093/humupd/dmr037 21987525

[10] Polyzos NP Devroey P. A Systematic Review of Randomized Trials for the Treatment of Poor Ovarian Responders: Is There Any Light At the End of the Tunnel? Fertil Steril (2011) 96(5):1058–61. 10.1016/j.fertnstert.2011.09.048 22036048

[11] Ferraretti AP La Marca A Fauser BC Tarlatzis B Nargund G Gianaroli L. Definition EwgoPOR: ESHRE Consensus on the Definition of ‘Poor Response’ to Ovarian Stimulation for In Vitro Fertilization: The Bologna Criteria. Hum Reprod (2011) 26(7):1616–24. 10.1093/humrep/der092 21505041

[12] Papathanasiou A. Implementing the ESHRE ‘Poor Responder’ Criteria in Research Studies: Methodological Implications. Hum Reprod (2014) 29(9):1835–8. 10.1093/humrep/deu135 24916434

[13] Younis JS Ben-Ami M Ben-Shlomo I. The Bologna Criteria for Poor Ovarian Response: A Contemporary Critical Appraisal. J Ovarian Res (2015) 8:76. 10.1186/s13048-015-0204-9 26577149 PMC4650906

[14] Poseidon G Alviggi C Andersen CY Buehler K Conforti A De Placido G. A New More Detailed Stratification of Low Responders to Ovarian Stimulation: From a Poor Ovarian Response to a Low Prognosis Concept. Fertil Steril (2016) 105(6):1452–3. 10.1016/j.fertnstert.2016.02.005 26921622

[15] Conforti A Esteves SC Picarelli S Iorio G Rania E Zullo F. Novel Approaches for Diagnosis and Management of Low Prognosis Patients in Assisted Reproductive Technology: The POSEIDON Concept. Panminerva Med (2019) 61(1):24–9. 10.23736/S0031-0808.18.03511-5 30021418

[16] Abu-Musa A Haahr T Humaidan P. Novel Physiology and Definition of Poor Ovarian Response; Clinical Recommendations. Int J Mol Sci (2020) 21(6):2110. 10.3390/ijms21062110 PMC7139860 32204404

[17] Xu Y Nisenblat V Lu C Li R Qiao J Zhen X. Pretreatment With Coenzyme Q10 Improves Ovarian Response and Embryo Quality in Low-Prognosis Young Women With Decreased Ovarian Reserve: A Randomized Controlled Trial. Reprod Biol Endocrinol (2018) 16(1):29. 10.1186/s12958-018-0343-0 29587861 PMC5870379

[18] Nagels HE Rishworth JR Siristatidis CS Kroon B. Androgens (Dehydroepiandrosterone or Testosterone) for Women Undergoing Assisted Reproduction. Cochrane Database Syst Rev (2015) 11):CD009749. 10.1002/14651858.CD009749.pub2 PMC10559340 26608695

[19] Haahr T Dosouto C Alviggi C Esteves SC Humaidan P. Management Strategies for POSEIDON Groups 3 and 4. Front Endocrinol (Lausanne) (2019) 10:614. 10.3389/fendo.2019.00614 31572298 PMC6749147

[20] Chen YX Ma K. Systematic Evaluation of Clinical Application of Dingkun Dan [in Chinese]. Zhongguo Zhong Yao Za Zhi (2015) 40(20):3916–9. 10.4268/cjcmm20152004 27062801

[21] Hou X Wan S. Study on Pharmacodynamics of Dingkundan Capsule [in Chinese]. J Shanxi Med Univ (2007) 38(12):1085–8. 10.1007/j.tcm.6611-1085.2007.12.04

[22] Song J-Y Gao D-D Sun Z-G. Efficacy and Safety of Ding-kun Pill Intervening Pregnancy Outcomes in Low-Prognosis Patients of Advanced Maternal Age Undergoing In Vitro Fertilization and Embryo Transfer [in Chinese]. Chin J Pract Gynecol Obstet (2020) 36(12):1200–4. 10.19538/j.fk2020120118

[23] Song J Ma T Liang Y Cao X Sun Z. Efficacy and Safety of Dingkun Pill for Female Infertility Patients With Low Prognosis Undergoing In Vitro Fertilization-Embryo Transfer: Study Protocol for a Multicenter, Double-Blind, Randomized, Placebo-Controlled Trial. Trials (2020) 21(1):550. 10.1186/s13063-020-04502-z 32560734 PMC7304132

[24] Dou XX Lin S Tian XH Zhang YH Guo X Ye J. Systematic Characterization of the Chemical Constituents In Vitro and Prototypes In Vivo of Dingkun Dan Using Ultra-High-Performance Liquid Chromatography Quadrupole Time-of-Flight Mass Spectrometry Combined With the UNIFI Software. BioMed Chromatogr (2020) 34(10):e4914. 10.1002/bmc.4914 32515056

[25] Gao X Wang N Jia J Wang P Zhang A Qin X. Chemical Profliling of Dingkun Dan by Ultra High Performance Liquid Chromatography Q Exactive Orbitrap High Resolution Mass Spectrometry. J Pharm BioMed Anal (2020) 177:112732. 10.1016/j.jpba.2019.06.029 31568965

[26] Brinkhaus B Pach D Ludtke R Willich SN. Who Controls the Placebo? Introducing a Placebo Quality Checklist for Pharmacological Trials. Contemp Clin Trials (2008) 29(2):149–56. 10.1016/j.cct.2007.06.005 17669693

[27] Haas J Zilberberg E Dar S Kedem A Machtinger R Orvieto R. Co-Administration of GnRH-Agonist and hCG for Final Oocyte Maturation (Double Trigger) in Patients With Low Number of Oocytes Retrieved Per Number of Preovulatory Follicles–A Preliminary Report. J Ovarian Res (2014) 7:77. 10.1186/1757-2215-7-77 25296696 PMC4237863

[28] Pan Y-F Lei J-Y Li X-R Meng C Xie X-Y. Tonifying Kidney, Soothing Liver and Harmonizing Blood Method Combined With Mild Stimulation Program in Treatment of Infertile Patients With Poor Ovarian Response [in Chinese]. Liaoning J Tradit Chin Med (2016) 43(7):1398–401. 10.13192/j.issn.1000-1719.2016.07.017

[29] Feng G-L You Z-L. Experience Summary of Professor Zhao-Ling You Treating POR With Bushen Jianpi Formula [in Chinese]. Chin Arch Tradit Chin Med (2012) 30(11):2379–81. 10.13193/j.archtcm.2012.11.13.fenggl.019

[30] Wei A-W Xiao H-D-Z Xu G-L Song Y-L. Clinical Outcomes of Dingkun Dan Combined With Micro Stimulation for Low Ovarian Response in IVF-ET [in Chinese]. Chin Arch Tradit Chin Med (2019) 37(9):2224–8. 10.13193/j.issn.1673-7717.2019.09.042

[31] Deng Y Wang YF Zhu SY Ma X Xue W Ma RL. Is There an Advantage of Using Dingkun Pill Alone or in Combination With Diane-35 for Management of Polycystic Ovary Syndrome? A Randomized Controlled Trial. Chin J Integr Med (2020) 26(12):883–9. 10.1007/s11655-020-3097-4 32915426

[32] Deng Y Xue W Wang YF Liu XH Zhu SY Ma X. Insulin Resistance in Polycystic Ovary Syndrome Improved by Chinese Medicine Dingkun Pill: A Randomized Controlled Clinical Trial. Chin J Integr Med (2019) 25(4):246–51. 10.1007/s11655-018-2947-1 31236888

[33] Xie M-Q. Effect of Dinkun Pills Combined With Clomiphene in the Treatment of Infertile Patients With Poor Ovarian Reserve [in Chinese]. Maternal Child Health Care China (2018) 33(23):5541–3. 10.7620/zgfybj.j.issn.1001-4411.2018.23.79

[34] Huang L Wang L Bao H Xu Y Meng M Qiao M. Traditional Chinese Medicine Dingkun Pill Facilitates Uterine Receptivity for Implantation in Micedagger. Biol Reprod (2019) 101(4):695–703. 10.1093/biolre/ioz141 31347662

[35] Ma K Chen Y Fan X Yuan Y Wang K Tian C. Dingkun Pill Replenishes Diminished Ovarian Reserve Through the PI3K/AKT/mTOR Signaling Pathway in TWP-induced Mice. J Ethnopharmacol (2020) 262:112993. 10.1016/j.jep.2020.112993 32473368

