# Supplementary figures and images for: The Role of Traditional Chinese Formula Ding-Kun Pill (DKP) in Expected Poor Ovarian Response Women (POSEIDON Group 4) Undergoing In Vitro Fertilization-Embryo Transfer: A Multicenter, Randomized, Double-Blind, Placebo-Controlled Trial
Source: Front Endocrinol (Lausanne). 2021 Jun 17;12:675997. doi: 10.3389/fendo.2021.675997 (PMC8247913; doi:10.3389/fendo.2021.675997)

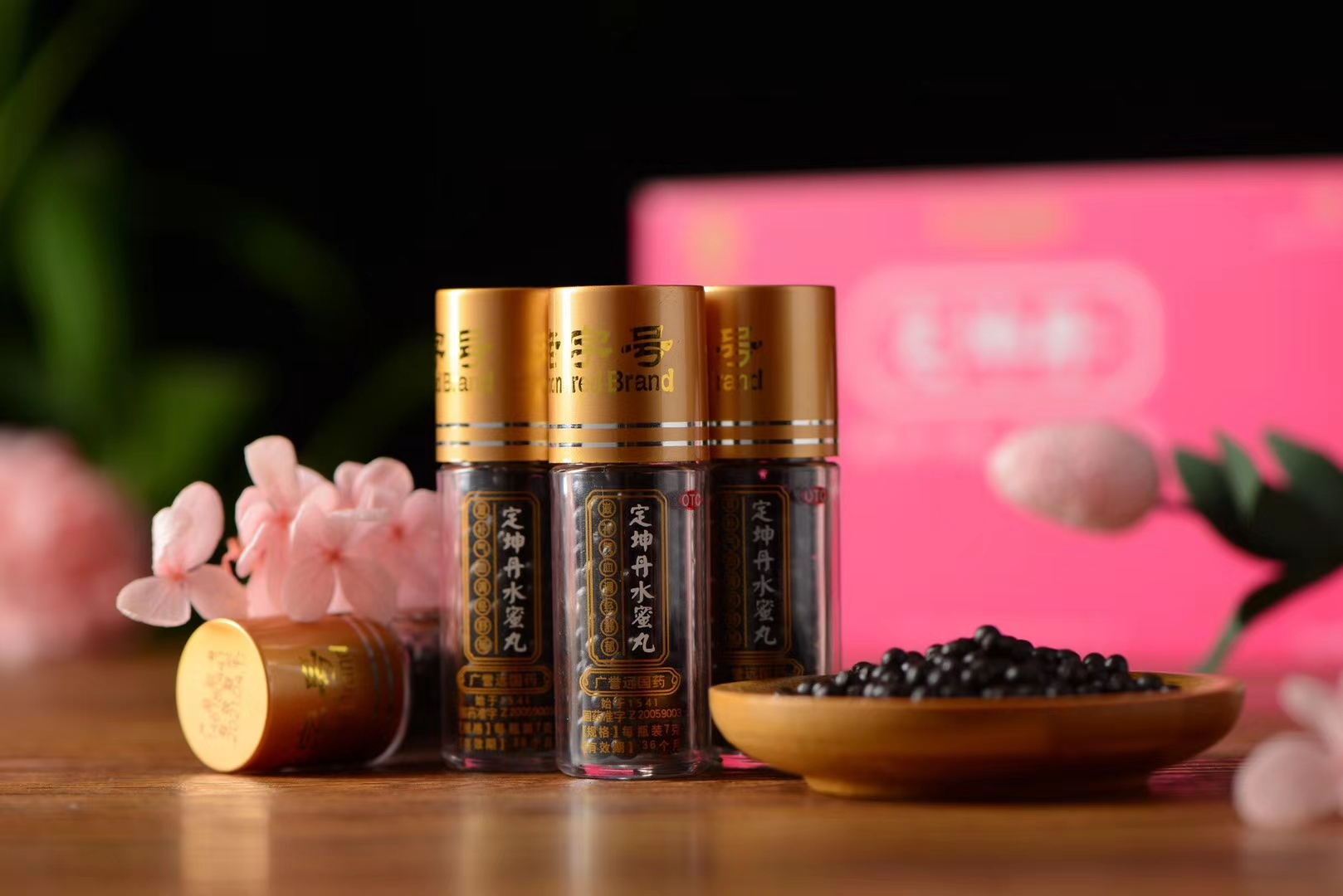

Supplement: Supplementary Figure 1 — Dingkun Pill, a traditional Chinese medicine with a long history. [file Image_1.tif]
