# Supplementary material for: The Role of Traditional Chinese Formula Ding-Kun Pill (DKP) in Expected Poor Ovarian Response Women (POSEIDON Group 4) Undergoing In Vitro Fertilization-Embryo Transfer: A Multicenter, Randomized, Double-Blind, Placebo-Controlled Trial
Source: Front Endocrinol (Lausanne). 2021 Jun 17;12:675997. doi: 10.3389/fendo.2021.675997 (PMC8247913; doi:10.3389/fendo.2021.675997)
Supplement: Supplementary file 2 [file DataSheet_1.docx]

| **eTable 1│Reproductive outcomes for women in Ding-Kun Pill and placebo groups (per protocol analysis). Data are number/total number (%) of women unless stated otherwise.** | | | | |
| --- | --- | --- | --- | --- |
| **Outcomes** | **Ding-Kun Pill group(n=224)** | **Placebo group (n=228)** | **Relative risk (95% CI)** | **P value** |
| **Primary outcome** |  |  |  |  |
| Ongoing pregnancy ⁕ |  |  |  |  |
| Ongoing pregnancy rate/No of randomised women | 61/224 (27.2) | 55/228 (24.1) | 1.13 (0.82 to 1.55) | 0.449 |
| Ongoing pregnancy rate/No of women who started stimulation | 61/224 (27.2) | 55/228 (24.1) | 1.13 (0.82 to 1.55) | 0.449 |
| Ongoing pregnancy rate/No of oocyte retrievals | 61/223 (27.4) | 55/225 (24.4) | 1.12 (0.82 to 1.53) | 0.482 |
| Ongoing pregnancy rate/No of embryo transfers | 61/206 (29.6) | 55/209 (26.3) | 1.13 (0.83 to 1.53) | 0.454 |
| **Secondary outcomes** |  |  |  |  |
| Clinical pregnancy |  |  |  |  |
| Clinical pregnancy rate/No of randomised women | 77/224 (34.4) | 69/228 (30.3) | 1.14 (0.87 to 1.48) | 0.350 |
| Clinical pregnancy rate/No of women who started stimulation | 77/224 (34.4) | 69/228 (30.3) | 1.14 (0.87 to 1.48) | 0.350 |
| Clinical pregnancy rate/No of oocyte retrievals | 77/223 (34.5) | 69/225 (30.7) | 1.13 (0.86 to 1.47) | 0.383 |
| Clinical pregnancy rate/No of embryo transfers | 77/206 (37.4) | 69/209 (33.0) | 1.21 (0.81 to 1.81) | 0.352 |
| Positive pregnancy † |  |  |  |  |
| Positive pregnancy rate/No of randomised women | 85/224 (37.9) | 81/228 (35.5) | 1.17 (0.84 to 1.36) | 0.594 |
| Positive pregnancy rate/No of women who started stimulation | 85/224 (37.9) | 81/228 (35.5) | 1.17 (0.84 to 1.36) | 0.594 |
| Positive pregnancy rate/No of oocyte retrievals | 85/223 (38.1) | 81/225 (36.0) | 1.06 (0.83 to 1.35) | 0.643 |
| Positive pregnancy rate/No of embryo transfers | 85/206 (41.3) | 81/209 (38.8) | 1.07 (0.84 to 1.35) | 0.602 |
| Pregnancy loss rate ‡ | 38/85 (44.7) | 36/81 (44.4) | 1.01 (0.72 to 1.41) | 0.973 |
| Pregnancy loss ≤ 12 weeks of gestation | 37/85 (43.5) | 34/81 (42.0) | 1.04 (0.73 to 1.48) | 0.840 |
| Pregnancy loss >12 weeks of gestation | 1/85 (1.2) | 2/81 (2.5) | 0.48 (0.04 to 5.15) | 0.614 |
| Ectopic pregnancies ‡ | 0/85 (0) | 1/81 (1.2) | — | 0.488 |
| Embryo implantation rate (median (IQR)) ⁋ | 0 (0.5) | 0 (0.5) | — | 0.500 |
| Twin pregnancies | 5/77 (6.5) | 11/69 (15.9) | 0.41 (0.15 to 1.11) | 0.068 |
| All analyses by per protocol.  ⁕ Ongoing pregnancy was defined as a detectable fetal heart beat after 20 weeks of gestation.  † Positive pregnancy (biochemical pregnancy), i.e. serum β-hCG level ≥ 10mIU/mL.  ‡ Denominator defined as number of positive β-hCG values (≥10 IU/mL) in each group.  ⁋ Embryo implantation rate was defined as the number of intrauterine gestational sacs observed divided by the number of embryos transferred. | | | | |
