# Supplementary material for: The Role of Traditional Chinese Formula Ding-Kun Pill (DKP) in Expected Poor Ovarian Response Women (POSEIDON Group 4) Undergoing In Vitro Fertilization-Embryo Transfer: A Multicenter, Randomized, Double-Blind, Placebo-Controlled Trial
Source: Front Endocrinol (Lausanne). 2021 Jun 17;12:675997. doi: 10.3389/fendo.2021.675997 (PMC8247913; doi:10.3389/fendo.2021.675997)
Supplement: Supplementary file 3 [file DataSheet_2.docx]

| **eTable 2│The definitions of secondary outcomes.** | |
| --- | --- |
| **Outcomes** | **Definitions** |
| Positive pregnancy rate | Positive pregnancy (biochemical pregnancy), i.e. serum β-hCG level ≥ 10mIU/mL, 14 days after embryo transfer. |
| Embryo implantation rate | Embryo implantation rate was defined as the number of intrauterine gestational sacs observed divided by the number of embryos transferred. |
| Clinical pregnancy rate | Clinical pregnancy, defined as an intrauterine gestational sac with fetal heartbeat detected by transvaginal ultrasonography after 6 weeks of gestation. |
| Ectopic pregnancy rate | Ectopic pregnancy, defined as a pregnancy in which implantation takes place outside the uterine cavity. |
| Pregnancy loss rate | Pregnancy loss, defined as clinically recognized spontaneous loss of pregnancy before the completion of twenty gestational weeks. |
| Twin pregnancies | A pregnancy in which two embryos develop in the uterus at the same time. |
