# Supplementary material for: The Role of Traditional Chinese Formula Ding-Kun Pill (DKP) in Expected Poor Ovarian Response Women (POSEIDON Group 4) Undergoing In Vitro Fertilization-Embryo Transfer: A Multicenter, Randomized, Double-Blind, Placebo-Controlled Trial
Source: Front Endocrinol (Lausanne). 2021 Jun 17;12:675997. doi: 10.3389/fendo.2021.675997 (PMC8247913; doi:10.3389/fendo.2021.675997)
Supplement: Supplementary file 4 [file DataSheet_3.docx]

| **eTable 3│Subgroup analysis of primary outcome (ongoing pregnancy rate per embryo transfer) for women in Ding-Kun Pill and placebo groups.** | | | | | | | |
| --- | --- | --- | --- | --- | --- | --- | --- |
| **Subgroups** | **Ding-Kun Pill group** | | **Placebo group** | | **Relative risk (95% CI)** | **P value** | **P value for**  **interaction** |
|  | **Total No.** | **Events (%)** | **Total No.** | **Events (%)** |  |  |  |
| Total | 206 | 61 (29.6) | 209 | 55 (26.3) | 1.13 (0.83 to 1.53) | 0.454 |  |
| Female age at oocyte retrieval |  |  |  |  |  |  | 0.028 |
| < 37 yrs. | 79 | 33 (41.8) | 71 | 18 (25.4) | 1.65 (1.02 to 2.65) | 0.034 |  |
| ≥ 37 yrs. | 127 | 28 (22.0) | 138 | 37 (26.8) | 0.82 (0.54 to 1.26) | 0.368 |  |
| Body mass index (BMI) |  |  |  |  |  |  | 0.394 |
| < 24 kg/m^2^ | 128 | 35 (27.3) | 137 | 37 (27.0) | 1.01 (0.68 to 1.50) | 0.951 |  |
| ≥ 24 kg/m^2^ | 78 | 26 (33.3) | 72 | 18 (25.0) | 1.33 (0.80 to 2.22) | 0.263 |  |
| FSH on menstrual cycle days 2-3 |  |  |  |  |  |  | 0.147 |
| ≤ 10 mIU/ml | 129 | 44 (34.1) | 124 | 32 (25.8) | 1.32 (0.90 to 1.94) | 0.150 |  |
| > 10 mIU/ml | 77 | 17 (22.1) | 85 | 23 (27.1) | 0.82 (0.47 to 1.41) | 0.463 |  |
| Embryo transfer stage |  |  |  |  |  |  | 0.402 |
| Cleavage stage | 178 | 48 (27.0) | 191 | 45 (23.6) | 1.15 (0.81 to 1.63) | 0.451 |  |
| Blastocyst stage | 28 | 13 (46.4) | 18 | 10 (55.6) | 0.84 (0.47 to 1.48) | 0.546 |  |
| No of embryos transferred |  |  |  |  |  |  | 0.624 |
| Single embryo transfer | 81 | 18 (22.2) | 82 | 18 (22.0) | 1.01 (0.57 to 1.80) | 0.967 |  |
| Double embryo transfer | 125 | 43 (34.4) | 127 | 37 (29.1) | 1.18 (0.82 to 1.70) | 0.369 |  |
| Embryo transfer protocol |  |  |  |  |  |  | 0.234 |
| Fresh embryo transfer | 106 | 32 (30.2) | 123 | 38 (30.9) | 0.98 (0.66 to 1.45) | 0.908 |  |
| Frozen-thawed embryo transfer | 100 | 29 (29.0) | 86 | 17 (19.8) | 1.47 (0.87 to 2.48) | 0.146 |  |
| Method of fertilization |  |  |  |  |  |  | 0.314 |
| IVF | 146 | 39 (26.7) | 155 | 41 (26.5) | 1.01 (0.69 to 1.47) | 0.959 |  |
| ICSI | 60 | 22 (36.7) | 54 | 14 (25.9) | 1.41 (0.81 to 2.48) | 0.218 |  |
| No. of top-quality embryos transferred |  |  |  |  |  |  | 0.177 |
| Yes | 77 | 29 (37.7) | 67 | 21 (31.3) | 1.20 (0.76 to 1.90) |  |  |
| No | 129 | 32 (24.8) | 142 | 34 (23.9) | 1.04 (0.68 to 1.58) |  |  |
| FSH = follicle stimulating hormone; IVF = in vitro fertilization; ICSI = intracytoplasmic sperm injection. | | | | | | | |
